# Calibration of Digital Holographic Camera for Bubble Gas Volumetric Flux Measurements

**DOI:** 10.3390/s25226969

**Published:** 2025-11-14

**Authors:** Victor Dyomin, Alexandra Davydova, Nikolay Kirillov, Igor Polovtsev

**Affiliations:** Laboratory for Radiophysical and Optical Methods of Environmental Research, National Research Tomsk State University, 36, Lenin Ave., Tomsk 634050, Russia; dyomin@mail.tsu.ru (V.D.); kns68@mail.tsu.ru (N.K.); polovcev_i@mail.ru (I.P.)

**Keywords:** digital holography, gas flow, calibration, bubbles, methane, Arctic seas, underwater measurements

## Abstract

**Highlights:**

**What are the main findings?**

- A method that allows determining the volumetric flux of the bubble gas flow based on the analysis of histograms of the cross-sectional areas of bubbles and their velocities measured on the basis of the holographic images of bubbles was developed.
- A calibration procedure for a digital holographic camera with calibration coefficient k = 2 in the gas volumetric flux range from 5 × 10^−4^ m^3^·m^−2^·s^−1^ to 15 × 10^−4^ m^3^·m^−2^·s^−1^ was described.

**What are the implications of the main findings?**

- The method can be applied to monitor weak gas emissions, including methane in the Arctic seas, and may be used for the calibration of acoustic sounding systems.
- The obtained results are confirmed by field data, which demonstrates the promising application of the method for environmental studies.

**Abstract:**

This study is aimed at developing and verifying a method that uses a digital holographic camera to measure the gas volumetric flux, which is relevant for the monitoring of gas emissions, in particular methane in the Arctic seas. The method is based on the analysis of histograms of cross-sectional areas of gas bubbles and their velocities obtained from holographic data. The result of the study is the determination of a constant calibration factor k = 2, taking into account the geometric factor of the camera and the deformation of the bubbles. The coefficient is determined in laboratory conditions, taking into account the area of the gas-generating site of a bubble generator simulating a gas flare. It is found that *k* remains stable in a wide range of a gas volumetric flux from 5 × 10^−4^ m^3^·m^−2^·s^−1^ to 15 × 10^−4^ m^3^·m^−2^·s^−1^ that limits the applicability of a working formula. Verification of the method in the field conditions of the Arctic expedition showed good agreement with the data obtained by the standard trap method: the discrepancy was only 5%. It was shown that the method is applicable for quantitative assessment of weak gas emissions, in particular methane, in the Arctic seas, where the measured volumetric fluxes are orders of magnitude lower than the established upper limit of the method.

## 1. Introduction

The measurement of gas flows in the aquatic environment is quite relevant for studying the global carbon cycle and climate change. In recent decades researchers have been paying special attention to the Arctic region, where intensive methane emissions from degrading subsea permafrost may severely impact the global climate [1,2]. Gas flares—flows of bubbles continuously rising from the bottom—are observed throughout the World Ocean, at depths ranging from several meters to several kilometers. The analysis of their composition showed that their main component is methane (CH_4_). The East Siberian Arctic shelf stores over 30% of the world’s methane and carbon dioxide reserves contained in bottom sediments under a layer of subsea permafrost [3,4]. In shallow areas of this shelf, the main path for the CH_4_ transfer from sediments to water is bubble transport [5]. In this regard, it is especially important to determine the volumes of methane that enters the water column and the atmosphere with rising bubbles [4].

The areas of the Arctic shelf with natural gas emissions can be detected by remote sensing [1,2,6]. One of the remote methods is laser spectroscopy; in particular, the method of laser-induced fluorescence (LIF) [6]. When seawater is irradiated with a laser, we can register spectral features that indirectly indicate the presence of methane. This method is useful for real-time anomaly search, but does not provide direct quantitative data on methane.

Such observations require sea truth measurements and calibrations. In situ contact sensors are used to directly measure the dissolved methane concentration. The most used and sensitive technology today is absorption spectroscopy with preliminary gas extraction. A special silicon membrane permeable to methane separates it from water, after which the gas concentration is measured in the air cavity using infrared detectors. Examples of such devices are the commercial systems METS (Franatech GmbH, Reppenstedt, Germany) and HydroC/CH_4_ (-4H-JENA engineering GmbH, Kiel, Germany), which can be installed on submersible vehicles or buoy stations [6,7]. Their main advantage is extremely high sensitivity, which allows detecting background concentrations of methane in the ocean. However, there is also a significant disadvantage: at low temperatures characteristic of the Arctic seas, the rate of gas diffusion through the membrane drops sharply, which significantly increases the response time of the sensor and makes the measurements complicated.

There are three basic methods to measure methane release gas flow during Arctic surveys: trap method [8], acoustic sounding [9,10,11,12,13,14,15] and direct measurements [16,17,18,19].

**Figure 1:**
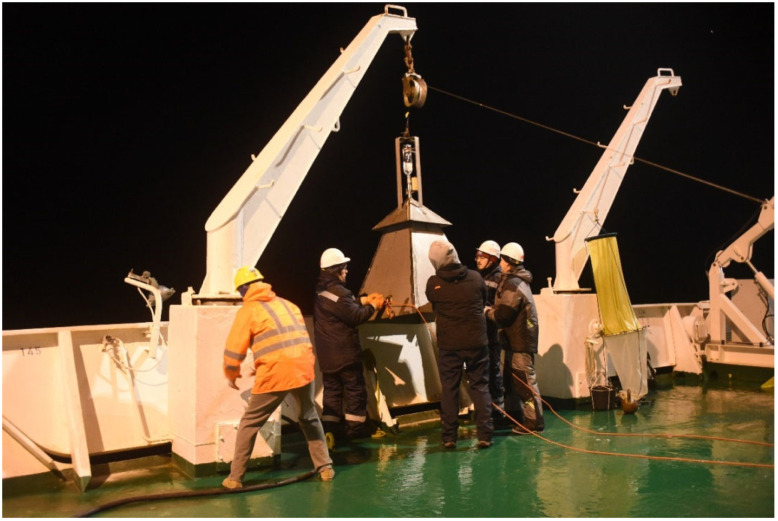
Installation of a gas-collecting bell for methane measurements during 82nd Arctic expedition on Akademik Mstislav Keldysh research vessel from 28 September to 4 November 2020.

Methane flow using a methane trap can be measured by installing a hollow bell with a gas-collecting bottle of fixed volume in the upper part of a probe above the water surface (Figure 1). The filling time of the bottle is determined by the gas flow rate from the surface. Calibrated values of bell opening area and bottle volume are the measurement parameters. The complexity of the measurement experiment is compensated in this case by direct measurements of the gas flow. But the stationary position of the measurement point does not confirm the spatial distribution of gas emissions.

**Figure 2:**
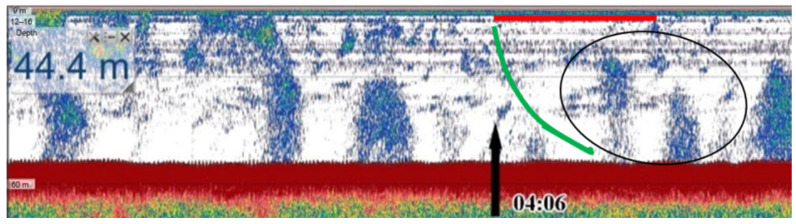
Echogram obtained during ship’s drift at station No. 6962 during 82nd Arctic expedition with simultaneous recording of holograms of gas bubble flow. Black arrow marks start of hologram recording. Oval shows a section of the water column with quite a few bubbles. Green line indicates estimated trajectory of a submersible digital holographic camera during drift, while red line shows desired trajectory of camera for correct interpretation of methane flow measurements [21].

Due to its large coverage area, acoustic sounding using standard or specialized echo sounders [20] and sonars [12] is the most effective and representative in terms of observing floating bubbles and obtaining estimates on the amount and spatial distribution of methane delivered by them to water and surface layers (Figure 2). Acoustic sounding is based on the fact that gas bubbles in the water column effectively scatter sound waves. A vessel equipped with a sonar system can map the so-called “gas flares”—columns of bubbles rising from the bottom—while in motion. However, the method does not provide information on the chemical composition of gas or its exact concentration in water, indicating only the presence of a bubble phase.

Such echograms require calibration to further become a measuring tool.

In field conditions, a submersible digital holographic camera can be used for calibration. It measures bubble parameters based on their holographic images. During the 82nd Arctic expedition we tested the simultaneous use of the holographic method and acoustic sounding [21]. Figure 2 shows some details of this experiment. However, this field experiment showed the need for a more accurate approach to the use of holographic data.

Digital holography is based on the recording of the interference pattern formed during the interaction of the reference wave (non-scattered laser radiation) and the object wave (radiation scattered on particles) [17,22,23,24,25]. The reconstruction of holograms makes it possible to obtain images of all particles, including gas bubbles, in the studied volume of water with a resolution of up to several microns [26,27,28,29,30].

Over the past decade, digital holography has found widespread use in marine studies, including:

- monitoring of plankton communities [18,20,31,32,33,34,35,36,37],
- study of suspended particles distribution [17,18,20,38,39,40],
- study of gas bubbles in the water column [19,20,41,42,43].

Modern research in the field of digital holography is aimed at increasing the processing speed of holograms through the use of machine learning and deep learning algorithms for automatic classification of particles [44,45,46,47,48]. In addition, there are works on the creation of holographic systems with extended depth of field [49] and the possibility of 3D particle tracking, which is especially important in studying the dynamics of bubbles [17,42,46]. These achievements open up new opportunities for more accurate and informative quantification of gas volumetric flux in situ, which will further improve the proposed methodology.

However, the use of a digital holographic camera to quantify gas flows, in turn, also requires special calibration techniques that consider both the features of the optical system and the specifics of the behavior of bubbles in an aqueous medium, which is the major focus of this article.

## 2. Materials and Methods

### 2.1. Experimental Unit and Software

**Figure 3:**
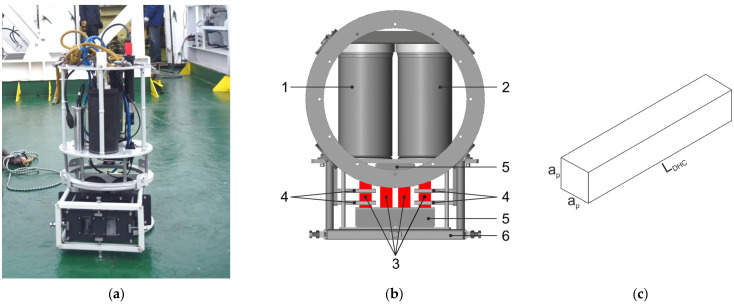
Submersible digital holographic camera DHC (**a**) and its scheme (**b**). 1—lighting unit, 2—recording unit, 3—studied medium volume (working volume), 4—calibers (test objects for calibration for magnification), 5—mirror-prism system to form a folded measuring channel in the medium (working volume) with length $L_{DHC}$, 6—welded frame. (**c**) Equivalent working volume of a folded measuring channel of the camera during a field experiment.

**Figure 4:**
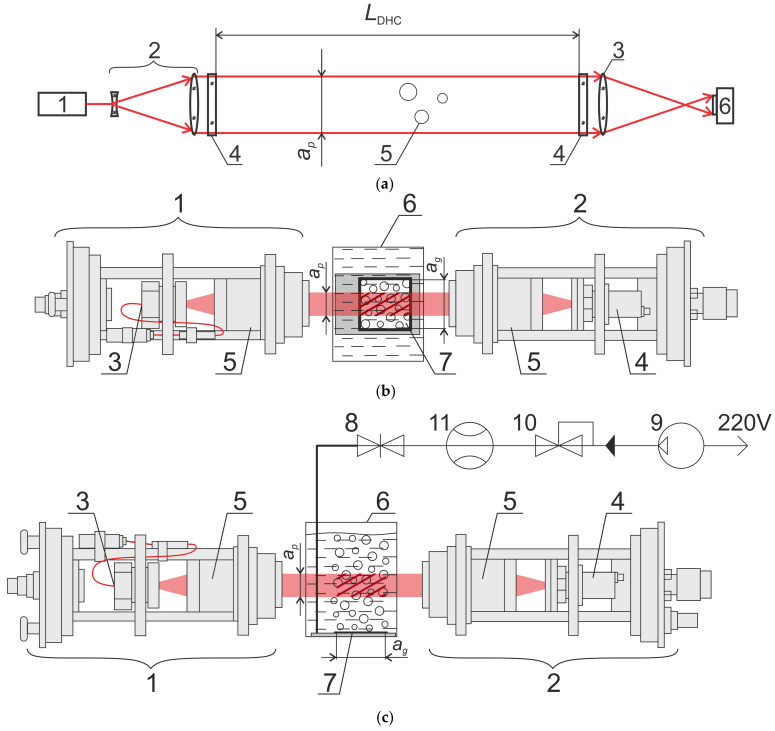
General scheme of in-line hologram recording: (**a**) 1—semiconductor laser diode, 2—beam expander, 3—receiving lens, 4—portholes, 5—bubbles, 6—CMOS camera. Scheme of methane flow measurements in a laboratory bench for calibration of a digital holographic camera with an artificial gas flare: (**b**) top view, (**c**) side view. 1—DHC lighting module, 2—DHC recording module, 3—fiber laser diode output, 4—CMOS camera, 5—collimating lenses, 6—cuvette with water, 7—gas bubble flow generating platform, 8—valve, 9—compressor, 10—pressure regulator, 11—flow meter, red area—collimated laser beam illuminating studied volume of water with bubbles. Red hatching indicates working volume with bubbles during calibration.

A submersible digital holographic camera (DHC) is used to record and measure gas bubbles in situ. In a number of works, such a camera was used to study plankton and settling particles [21] both in the monitoring mode at a fixed station and in the vertical profiling mode on a research vessel. Figure 3 shows the DHC with horizontally oriented working volume, which ensures vertical scanning of the water column for free flow of particles and bubbles in the vertical direction through the working volume of the camera. The main parts of the DHC are the lighting (1) and recording (2) units, which include the laser lighting module and the recording module (further shown in Figure 4) connected by a synchronization line. Four calibration test objects (4) [50] are installed in the working volume of the camera in the path of a laser beam for calibration for magnification. Strong deep-water cases of units (1) and (2) with portholes and connectors are connected by a frame (6) with base surfaces for interchangeable assembly. All cases and other DHC devices are electrically connected through sealed connectors and marine cables.

The DHC lighting and recording modules were used in the laboratory calibration experiment (Figure 4). Figure 4a shows the general scheme of in-line hologram recording, which is used both in a submersible device (Figure 3) and in a laboratory experiment. The lighting module has a laser diode (λ = 650 nm, P = 50 mW) and a synchronization system, while the recording module has a CMOS camera (resolution—2048 × 2048 pixels, pixel size—5.5 microns, frame rate—24 fps). Due to the square matrix, the entrance pupil of the camera is also square (size—$a_{p}$ × $a_{p}$). The synchronization system ensures the desired laser operating mode with adjustable pulse duration and time span to measure bubble velocity.

Figure 3c shows a drawing of an equivalent working volume of a folded measuring channel with bubbles recorded per one exposure on one hologram during a marine experiment with the DHC to measure gas flow under natural conditions. The drawing shows that in the vertical plane this volume is limited by the entrance pupil, and by the length of the measuring channel $L_{DHC}$ in the direction of the optical axis located horizontally.

The DHC working volume used for field measurements was $V_{DHC}=0.75$ dm^3^.

A special stand was developed for laboratory calibration experiments. It includes the following main components (Figure 4): a bubble generator, lighting and recording modules of the DHC and a cuvette with water.

**Figure 5:**
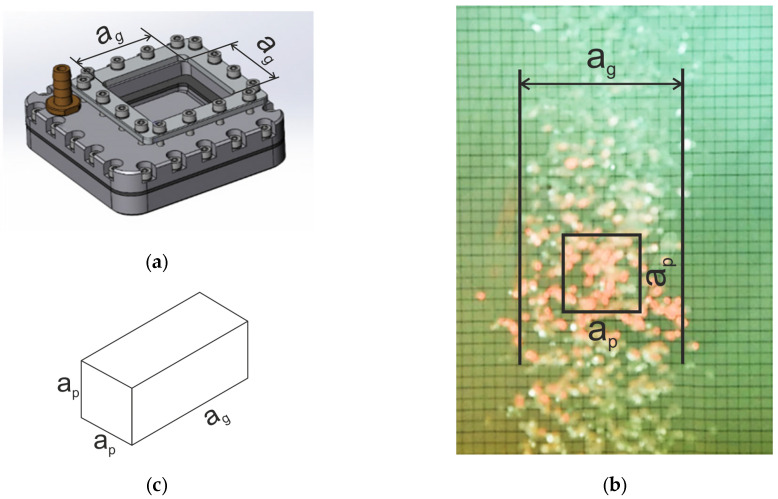
Bubble flow generating platform $a_{g}\times a_{g}$ (**a**) and a “uniform” flow formed by it against background of entrance pupil of a digital holographic camera with size $a_{p}\times a_{p}$ (**b**). (**c**) Measuring volume during calibration.

The bubble generator is arranged in such a way that the created flow is concentrated inside the generating platform—a perforated plate with an area of $S_{g}=a_{g}\times a_{g}=100\times 100{\mathrm{mm}}^{2}$ (Figure 5a). The size of the entrance pupil side in the recording module $a_{p}$ is slightly smaller than $a_{g}$, which makes it possible to arrange the generating platform so that the optical axis of the DHC passes in a place of a uniform flow of bubbles (Figure 5b). In addition to the generating plate (7) placed on the cuvette bottom, the generator includes start-up and control devices: a valve (8), a compressor (9), a pressure regulator (10) and a flow meter (11), which allows measuring gas flow rate when bubbles are generated.

Figure 5c shows a drawing of the working volume with bubbles (shown by red hatching in Figure 4) recorded per one exposure on one hologram during a laboratory experiment with a bubble generator. It demonstrates that in the vertical plane this volume is limited by the entrance pupil with the size $a_{p}\times a_{p}$, and by the side of the generating platform $a_{g}$ in the direction of the optical axis located horizontally.

The holographic principle of bubble recording is the same for both the laboratory experiment (Figure 4) and the marine experiment (Figure 3) and is described below. A collimated beam of the laser diode-induced coherent radiation illuminates the water medium volume with bubbles. Hence, light scattering on particles (bubbles) causes an object wave field, which, in turn, interacts with an illuminating field not scattered on particles, and forms an interference pattern. The CMOS camera matrix registers this interference pattern as a two-dimensional array of intensities representing a digital hologram of the working volume. The software makes it possible to obtain sharp images of all transverse layers of the recorded volume and all particles located at different distances in the measuring volume at the stage of recording of one particular hologram. Images are reconstructed from a digital hologram numerically by calculating the complex amplitude in the corresponding volume section using the diffraction integral [22,23]:

(1) $$U\left(x_{2},y_{2},z\right)=\int_{-\infty }^{\infty }\int_{\infty }^{\infty }I_{H}\left(x_{1},y_{1}\right)\cdot \frac{exp\left(ikz\right)}{i\lambda z}\cdot exp\left\{\frac{ik}{2z}\left[{\left(x_{2}-x_{1}\right)}^{2}+{\left(y_{2}-y_{1}\right)}^{2}\right]\right\}dx_{1}dy_{1},$$

where $U\left(x_{2},y_{2},z\right)$ is the complex amplitude of the reconstructed field in the plane $\left(x_{2},y_{2}\right)$ at a distance z from the hologram plane $\left(x_{1},y_{1}\right)$, $I_{H}\left(x_{1},y_{1}\right)$ is the intensity distribution recorded by the CMOS camera, λ is the wavelength and k=2πλ is the wave number.

The task of reconstructing a particle image can be addressed by the fact that for each particle of the volume there is such a value z, which satisfies the conditions of focusing (the best image, i.e., the sharpest image), formulated in relation to the intensity $I\left(x_{2},y_{2},z\right)={\left|U\left(x_{2},y_{2},z\right)\right|}^{2}$. At the same time, we observe the loss of information on the phase of a light wave, which prevents the reconstruction of a 3D shape of each particle, but do not observe the problem of ‘phase unwrapping’ [51,52,53,54] associated with the cyclic nature of phase measurements in the range [−π, π]. However, in our case, it is not necessary to determine the 3D shape of the particle (bubble) surface, since we work with the image of its central section, and the boundary contrast criterion [49] is sufficient enough to determine the boundary of the particle image and its cross-section area. The direct calculation of the diffraction integral (1) is quite time consuming; therefore, we used a method based on the application of the convolution theorem [23]. The image reconstructed from the digital hologram is the intensity distribution of the complex amplitude of the reconstructed field, from which the intensity distribution $I\left(x_{2},y_{2},z\right)={\left|U\left(x_{2},y_{2},z\right)\right|}^{2}\;$ is obtained. The geometric parameters (area, size) of a particle section are measured directly from the image in the best image plane [55]. The reconstructed image size is 2048 × 2048 pixels, and the pixel size is determined using calibration test objects [50] for each experiment.

For simultaneous post-processing, such sharp images of particles from different layers are aligned in one plane and form a 2D display of a holographic image of the studied volume with bubbles and, if present, other particles [55].

Subsequent processing of the 2D display using the DHC software (V1.2) involves binarization and automatic extraction of particle images. The selected images of particles are used to determine their geometric parameters and coordinates of the center of gravity, and the automatic classification based on the morphological feature [55] makes it possible to classify the “Bubbles” taxon. Thus, we determine the size $H_{i}$ for each *i* bubble in the horizontal direction and the cross-sectional area $S_{i}$ in the vertical plane [55].

**Figure 6:**
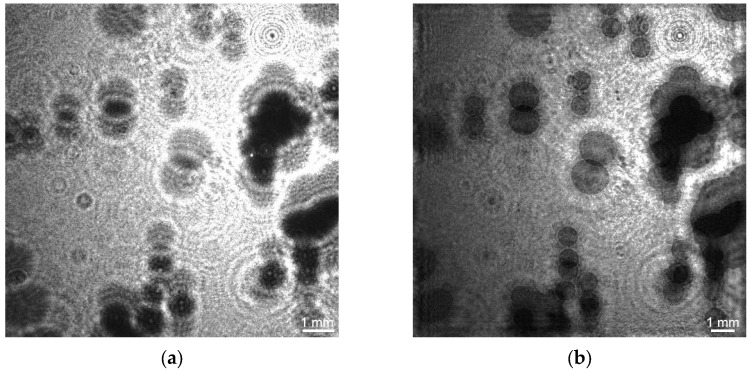
A digital hologram obtained by superimposition of two holograms of helium bubbles shifted in time by 2500 μs (**a**) and a 2D display of a holographic image of water volume with helium bubbles (**b**) obtained therefrom for a gas flow—2.83 × 10^−6^ m^3^/s.

**Figure 7:**
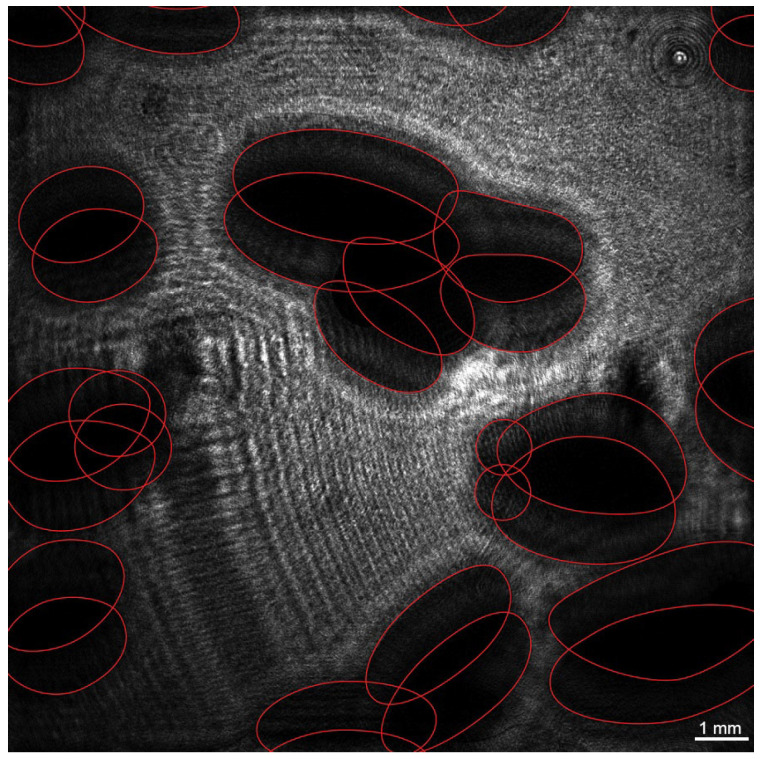
2D display of a holographic image of water volume with air bubbles, obtained from superimposed holograms recorded with a time shift of 2500 μs for a flow of 2.83 × 10^−6^ m^3^/s. Red ellipses show two adjacent positions of the bubble. Ellipses limit *S_i_*—cross-sectional area of bubble in vertical plane, *H_i_*—larger size.

The superimposition of holograms was used to measure the vertical component of bubble velocity (hereinafter referred to as bubble velocity) when the recorded hologram represents the superimposition of two holograms separated by a time interval (Figure 6a). To identify the two positions of one bubble, it is necessary that the bubble does not move a distance greater than its size, which requires an appropriate time resolution not provided by the CMOS frame rate of the camera. This problem was solved through two-pulse lighting using a synchronizing device. Figure 6b shows a two-dimensional display of a holographic image of the water volume with helium bubbles, and Figure 7 with air bubbles. The red ellipses in Figure 7 show two adjacent positions of bubbles.

**Figure 8:**
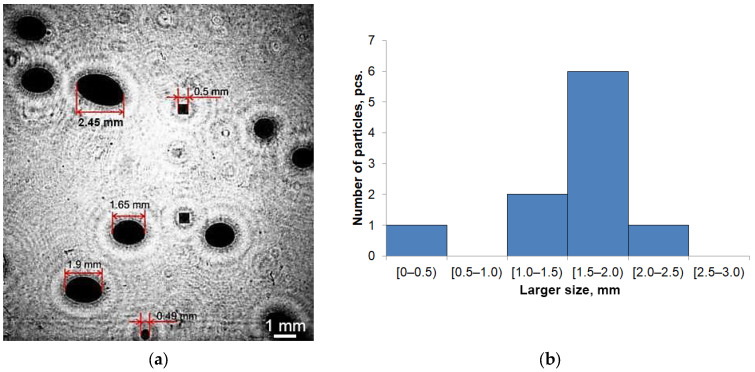
2D display of a holographic image of the studied volume with bubbles against background of model calibration particles (**a**) and a distribution histogram of bubbles according to larger size (**b**).

The calibration of the DHC magnification was performed in accordance with the procedure described in [50]: the sizes of bubbles studied in the laboratory experiment were determined by comparing the size of the images of bubbles with the known size of test particles (model particles), and a distribution histogram was compiled according to the larger size of bubbles (Figure 8). For the bubbles shown in the figure the larger size, $H_{i}$ was 2.45 ± 0.02 mm, 1.65 ± 0.02 mm, 1.90 ± 0.03 mm, 0.49 ± 0.01 mm, 0.50 ± 0.01 mm. The bubble size error averaged 1.5%.

### 2.2. Bubble Gas Volumetric Flux Measuring Theory and Mathematical Tools

The basic principle of measuring the volumetric gas flow (Q) using digital holography is that the method allows recording and measuring the key parameters of each bubble in the controlled volume of water: its cross-sectional area in the vertical plane ($S_{i}$) and speed in the vertical direction ($v_{i}$). The total contribution of all bubbles that passed through the measuring section during the exposure time gives the desired volumetric flow. Below are the main calculation formulas connecting the values $S_{i}$ and $v_{i}$ directly measured by the holographic method with the volumetric flow of bubble gas.

By definition, the volumetric gas flow rate Q is expressed by the formula $Q=\frac{V}{t}$, where V is the volume of gas flowing through the flow cross-section during time t. If we assume that the cross-sectional area of the flow is S, and during time t the flow passes the path l, then we can write Q=S·v, where $v=\frac{l}{t}$—gas flow rate.

When the methane trap is used, we measure the parameter [56] called the gas volumetric flux

(2) $$p=\frac{Q}{S}=\frac{V}{t\cdot S}.$$

In this case V can be measured in dm^3^, t in hours (minutes, seconds, days), S in square meters.

The volumetric gas flow carried by each spheroid bubble in the holographic experiment will be as follows:

(3) Qi=16πHi2·lit=16πHi2·hi·lit·hi=16Si·Hi·lit·hi,

where $l_{i}$ is the path passed by a bubble during time *t* in the vertical direction coinciding with the minor axis of the spheroid with length $h_{i}$ and $S_{i}$ is the cross-sectional area of the bubble in the vertical plane.

Volumetric gas flow transferred by all n bubbles of the working volume registered on the hologram in a particular experiment will be as follows:

(4) Qh=∑i=0n16kdi·Si·vi,

where $k_{di}=\frac{H_{i}}{h_{i}}$ is the deformation coefficient of the ellipse (inverse to the compression coefficient) in the vertical plane.

In accordance with Formula (2), let us write the following for the gas volumetric flux:

(5) $$p_{h}=\frac{Q_{h}}{S_{h}},$$

where $S_{h}$ is the area of the horizontal section of the camera working volume containing bubbles and recorded by a hologram. At the same time, in accordance with Figure 3c, $S_{h}=a_{p}\cdot L_{DHC}\;$ during natural marine measurements (provided that the transverse size of the bubble gas fountain is greater than $L_{DHC}$), and during calibration measurements in laboratory experiments in accordance with Figure 5c—$S_{h}=a_{p}\cdot a_{g}.$ According to Formula (2), the volumetric flux of the generated gas flow through the generating site (Figure 5) is

(6) $$p_{g}=\frac{Q_{g}}{S_{g}},$$

where $Q_{g}$ (m^3^/s) is the volumetric gas flow rate recorded using the flow meter, $S_{g}={a_{g}}^{2}=100^{2}\;$ mm^2^ for the described experimental scheme (Figure 5) and $a_{g}=100$ mm for the side of the gas-generating site.

Since for the volumetric flux registered with a digital holographic camera during calibration in laboratory conditions from Formulas (4) and (5) it is possible to write the following:

(7) phk=∑i=0n16kdi·Si·viap·ag

and the condition of uniform density of the flow generated by the gas-generating site is structurally provided, we can write $p_{g}=\;p_{hk}\;$ and then, from (6) and (7) for the volumetric flow rate of gas during calibration, we obtain the formula

(8) Qh=16ag∑i=0nkdi·Si·viap≅k∑i=0nSi·vi.

Here it is assumed that the degree of deformation of spheroids $k_{di}≅k_{d}$ is approximately the same for all bubbles, which explains the sign ≅. Then, k= 16ag kdap can be interpreted as a calibration factor for a given holographic camera and for specific experimental conditions.

Then, in accordance with Formula (5), the volumetric flux recorded during a natural marine experiment taking into account the results of laboratory calibration and the results of natural measurements of the parameters of bubbles $S_{i}\;$ and $v_{i}$ will be determined as follows:

(9) $$p_{s}=\frac{k}{a_{p}\cdot L_{DHC}}\sum_{i=0}^{n}S_{i}\cdot v_{i}.$$

Expression (9) is used to measure the gas flow in situ based on the digital holographic data obtained in the updated DHC software for processing the Bubbles taxon data, which involves measuring $S_{i}\;$ and $v_{i}$ for each i bubble.

Then, the calibration task in the laboratory experiment is to determine the coefficient *k* based on the Formula (6):

(10) $$k=\frac{Q_{g}}{\sum_{i=0}^{n}S_{i}\cdot v_{i}}.$$

The main tasks of such an experiment are as follows:

1. How constant is *k* and what is this coefficient for bubbles with different formation conditions (formed by different gases, at different volumetric flux and for different water salinity)?
2. In what range of the volumetric flux (gas flow rates) does Formula (7) apply?

### 2.3. Measurement Technique

Two gases (helium and air) and two types of water (clean and salt) were used in the laboratory experiment. Calibration tests were performed in the following sequence:

1. Setting a specified gas flow rate through the bubble generator
2. Recording a series of holograms (at least 100 dual holograms in each bubble generator mode)
3. Reconstructing bubble images from holograms
4. Determining for each bubble:
  - Area of a vertical bubble cross-section—(*S_i_*)
  - Speed *v_i_*—according to a shift on superimposed holograms
5. Calculating the total gas flow from DHC data
6. Comparing with the specified gas flow rate to determine the calibration factor using Formula (10).

The average data are presented in the format mean $\pm \frac{SD}{\sqrt{\Lambda }}$. Here, *t* is Student’s coefficients for the significance level ≤ 0.05 and the number of samples (holograms) Λ, and SD is the normal deviation.

### 2.4. Natural Measurements

The possibilities and features of methane bubbles using the holographic method were tested in natural conditions during the 2020 Arctic expedition [21].

**Figure 9:**
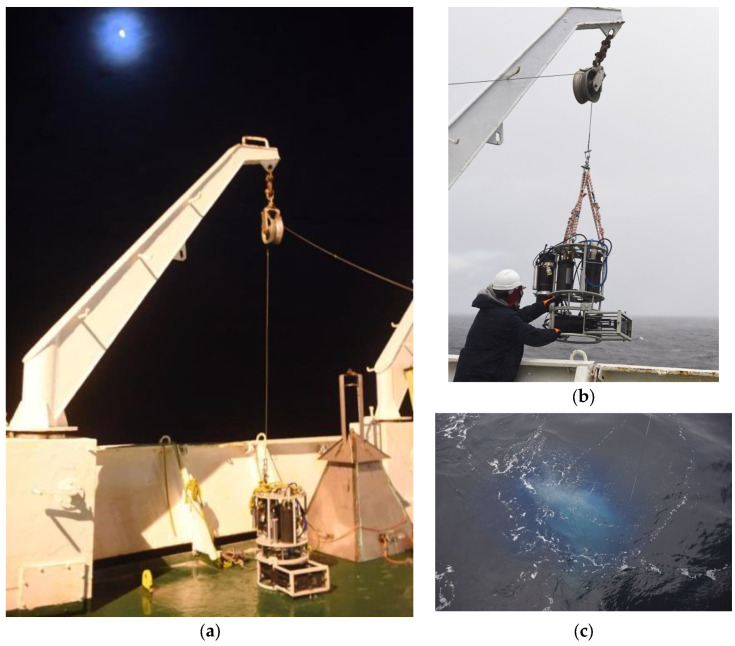
Gas-collecting bell (right) and DHC on AMK-82 deck (**a**). (**b**) DHC dive. (**c**) Methane release.

For this, the same methane flow from the methane sieve was measured using a gas-collecting bell and the DHC (Figure 9).

Natural measurements were used to compare the data using a methane trap at stations No. 6964 and No. 6975 on the AMK-82.

## 3. Results

### 3.1. Bubble Flow Characteristics

**Figure 10:**
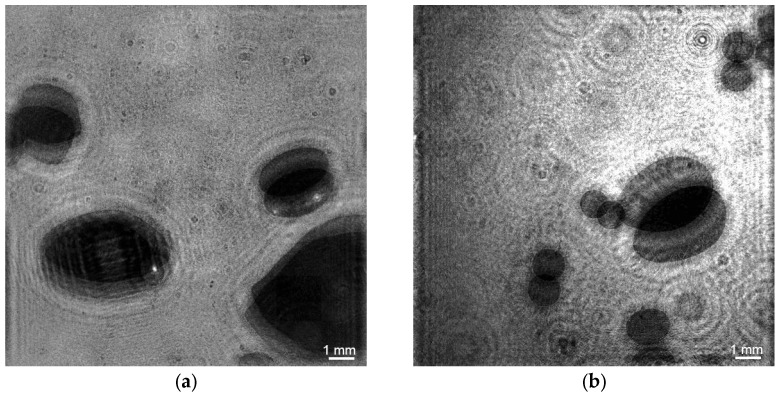
2D display of a double holographic image of studied volume with air (**a**) and helium (**b**) bubbles at a gas flow rate of 2.8 × 10^−6^ m^3^/s.

In laboratory experiments the analysis of reconstructed images made it possible to obtain the detailed bubble flow characteristics. The bubbles remain spherical when their diameters are quite small (~1 mm). Otherwise, the bubbles begin to deform into a flattened spheroid [57]. This is especially evident for air bubbles (Figure 10a).

**Figure 11:**
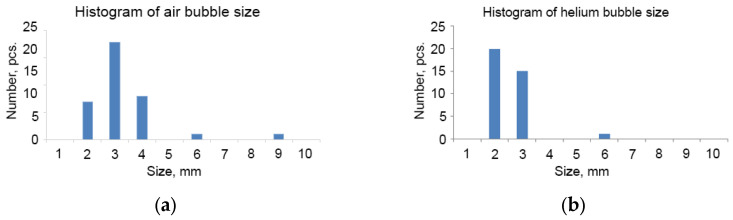
Histograms of larger dimensions of air (**a**) and helium (**b**) bubbles at same gas flow rate (2.83 × 10^−6^ m^3^/s).

With the same gas flow rate of 2.83 × 10^−6^ m^3^/s, smaller bubbles prevail in the flow of helium bubbles compared to the flow of air bubbles (Figure 10 and Figure 11). The average larger size of helium bubbles is 2.1 ± 0.2 mm and air bubbles is 2.7 ± 0.4 mm.

**Figure 12:**
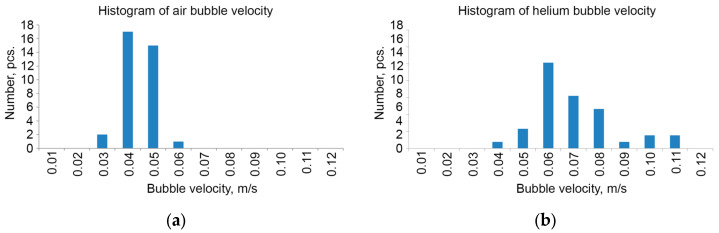
Histograms of velocities of air (**a**) and helium (**b**) bubbles.

The velocity of helium bubbles is greater than the velocity of air bubbles at the same gas flow rate (2.83 × 10^−6^ m^3^/s), which is confirmed by the velocity histogram (Figure 12). The average velocity of helium bubbles is 0.068 ± 0.009 m/s and air bubbles is 0.039 ± 0.008 m/s.

**Figure 13:**
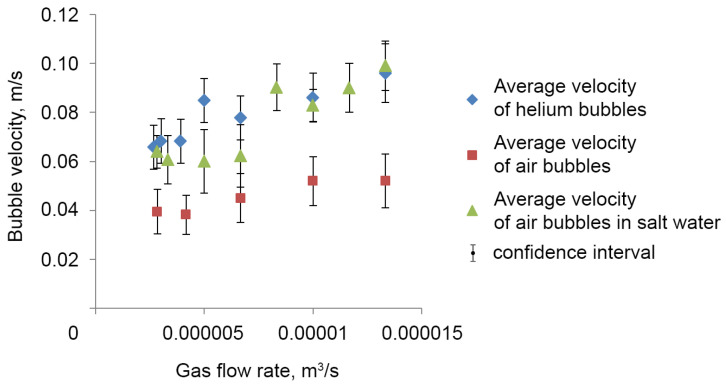
Bubble velocity versus gas flow rate.

The average bubble velocity increases with an increase in gas flow rate (Figure 13).

The histograms show that changes in the size of bubbles due to a change in gas and medium are compensated by a change in speed, which leads to a constant coefficient k.

**Figure 14:**
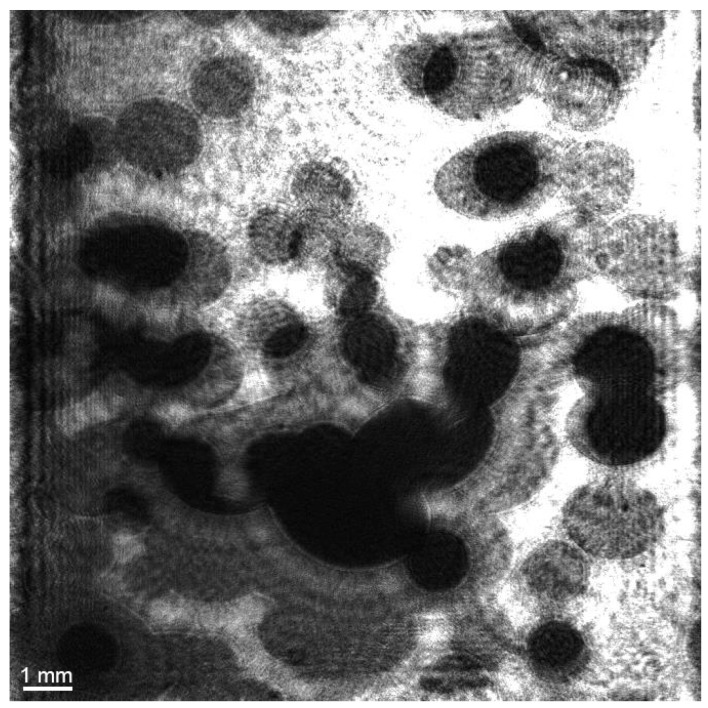
Image of helium bubbles at a gas flow rate of 15 × 10^−6^ m^3^/s, reconstructed from a digital hologram at a distance of 63 mm from surface of a matrix recording a digital hologram.

When the gas flow rate reaches about 15 × 10^−6^ m^3^/s, this fosters superimpositions and shielding of bubbles with each other (or the second position of a bubble is out of sight, so its velocity cannot be determined). Figure 14 shows an example of an image of helium bubbles at a gas flow rate of 15 × 10^−6^ m^3^/s. The image is reconstructed from a digital hologram at a distance of 63 mm from the plane of a matrix recording a digital hologram.

### 3.2. Calibration Dependencies

**Figure 15:**
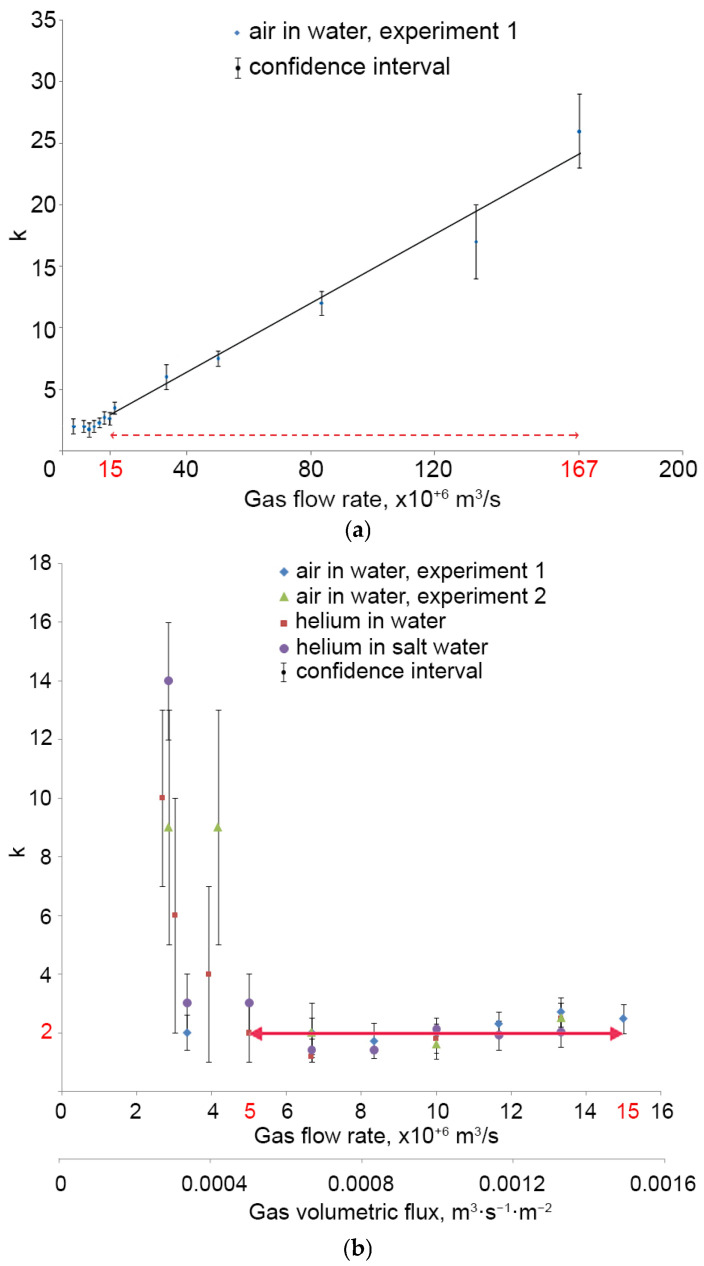
Calibration coefficient versus gas flow rate. Red arrow shows applicability range of working formula. Solid red arrow on (**b**) shows main measurement range, while dashed red arrow on (**a**) shows additional measurement range.

A series of experiments made it possible to establish the dependence of the calibration coefficient *k* on the gas flow rate in clean and salt water (Figure corresponds to the graph “air in water, experiment 1” in Figure 15b). Thus, *k* sharply increases with an air flow rate above 15 × 10^−6^ m^3^/s, which is associated with the effects of bubble overlapping and shielding (Figure 7). Within the air flow rate from 2.5 × 10^−6^ to 15 × 10^−6^ m^3^/s, the coefficient *k* remains *k* = 2.2 ± 0.5.

Figure 15b shows the dependence of the coefficient *k* on gas flow rate: for air in clean water (two experiments “air in water, experiment 1” and “air in water, experiment 2” for different cameras with different magnification calibrations), for helium in clean water (“helium in water”) and in salt solution (“helium in salt water”). Within the gas flow rate from 5 × 10^−6^ to 15 × 10^−6^ m^3^/s, the coefficient *k* remains almost constant: for air in clean water k = 2.0 ± 0.5, for helium in clean water k = 1.9 ± 0.5, for helium in salt solution k = 2.0 ± 0.5. Within the confidence interval, the calibration coefficient *k* remains constant for air and helium (including clean water and salt solution) and is taken equal to 2. The guaranteed relative error of the measured value is 25%. Hence, the specified gas flow range and the corresponding gas volumetric flux range is the main measurement range for the calibrated DHC.

The above error is random. We associate the causes of its occurrence with the discreteness and non-laminarity of the flow, as well as by the fact that some bubbles are cut by the entrance pupil of the DHC (Figure 6). If the first two reasons are due to the object of measurement, the latter can be reduced by optimizing the DHC design in the direction of increasing the aperture. Similar errors (−30%) for methane bubbles were obtained in [58] using direct measurements of methane concentrations in water samples taken using a bathometer and studied using the methods of spectral analysis. The authors of this work explain the high error by the high spatial variability of the analyzed flow.

Few bubbles are generated with low gas flow rate, which leads to high flow non-uniformity. For example, 27 out of 100 holograms do not contain bubbles with the gas flow rate of 2.5 × 10^−6^ m^3^/s, while the rest contain only one or two bubbles. This leads to low accuracy (relative error from 30% to 67%) of coefficient *k* within the gas flow rate from 2.5 × 10^−6^ to 5 × 10^−6^ m^3^/s.

Figure 15 shows that the calibration coefficient (k = 2) is a constant and does not depend on the measured gas flow with gas flow rate from $Q_{g}=$ 5 × 10^−6^ m^3^/s and no more than $Q_{g}=15\cdot 10^{-6}$ m^3^/s, which corresponds to the measured volumetric flux ranging from 5 × 10^−4^ m^3^·m^−2^·s^−1^ to 15 × 10^−4^ m^3^·m^−2^·s^−1^. Therefore, when determined in laboratory conditions, the calibration coefficient can be used for measurements in the water area using Formula (10) if the changes in the measured parameter fit within these basic range constraints.

The expansion of the measurement range towards smaller values of the gas volumetric flux is undoubtedly possible. However, in this case, the measurement error is not guaranteed and significantly exceeds the basic one.

The expansion of the measurement range towards larger values of the gas volumetric flux is also possible, but then it is necessary to take into account the almost linear growth of the coefficient k shown in Figure 15a. This linearity is caused by the fact that the large number of bubbles leads to their superimposition, and hence, we face the scenario when we “miss” the obscured part while calculating the areas of bubbles. Figure 15a shows the course of the approximation line and the corresponding expanded measurement range, which guarantees the basic error provided that the linearity *k* is taken into account.

### 3.3. Comparison with Natural Data

Natural data for station No. 6964 on the AMK-82 were used to compare the data (Table 1). Here, the methane flow from an area of 1 m^2^ was measured by the sounding method. At different times of the day, the volumetric flux was (according to the data given in [21]): p = 11 L/m^2^/day ≈ 0.13 × 10^−6^ m^3^·m^−2^·s^−1^ and p > 108 L/m^2^/day ≈ 1.5 × 10^−6^ m^3^·m^−2^·s^−1^. For gas flow rate of $Q_{g}$ = 16 × 10^−6^ m^3^/s, through a gas-generating platform $S_{g}$ = 10^4^ mm^2^, we get gas volumetric flux in the calibration experiment *p_g_* = 16 × 10^−4^ m^3^·m^−2^·s^−1^, which is significantly higher than the volumetric flux recorded at the station, which guarantees the working measurement range of a holographic camera near much stronger gas emissions.

The comparison of the volumetric flux measurements using a submersible holographic camera and the trap method at stations with a strong gas-hydrate emission near station No. 6975 showed a good result with an error of 5%, which confirms the efficiency of the technique.

## 4. Conclusions

A method of using a digital holographic camera to measure the volumetric flux of a bubble gas flow was developed and verified.

Histograms of bubble cross-sectional areas and their velocities determined from holographic data are used to calculate the volumetric flux.

A calibration technique was developed where the coefficient *k*, taking into account the geometric parameters of the digital holographic camera and the degree of deformation of bubbles, is determined in laboratory conditions, taking into account the area of the gas-generating site of the bubble generator used to create a model gas flare. At the same time, the range of action of the working formula for gas volumetric flux is limited by gas flow ranging from 5 × 10^−4^ m^3^·m^−2^·s^−1^ to 15 × 10^−4^ m^3^·m^−2^·s^−1^.

In natural conditions of the Arctic expedition, the volumetric flux of the methane flow measured by a standard method using a gas-collecting bell were compared with the proposed method using a submersible digital holographic camera. The match was within 5%. The volumetric flux typical of natural conditions in the Arctic seas turned out to be orders of magnitude lower than the working range of the proposed method, which indicates its applicability for the monitoring of methane emissions in the Arctic seas.

## Figures and Tables

**Table 1:** Comparison of gas flow characteristics.

Parameter	Laboratory DHC Data, Air	Laboratory DHC Data, Helium	Field Data, Methane, Trap Method, Weak Release, Station 6964	Field Data, Methane, Strong Release, Station 6975	
				DHC	Trap Method
Volumetric flux, m^3^·m^−2^·s^−1^	2.5 × 10^−4^–15 × 10^−4^	2.5 × 10^−4^–15 × 10^−4^	0.13 × 10^−6^–1.5 × 10^−6^	9.8 × 10^−4^	10 × 10^−4^
Mean bubble diameter, mm	2.7 ± 0.4	2.1 ± 0.2	N/A	2.2 ± 0.6	N/A
Mean bubble velocity, m/s	0.039 ± 0.008	0.068 ± 0.009	N/A	0.05 ± 0.01	N/A

N/A—not applicable.

## Data Availability

The data presented in this study are available on request from the corresponding author.

## Abbreviations

The following abbreviations are used in this manuscript:

DHC	Digital Holographic Camera
AMK-82	82nd Arctic expedition on the research vessel Akademik Mstislav Keldysh
